# Urinary Angiotensinogen Is Elevated in Patients with Nephrolithiasis

**DOI:** 10.1155/2014/349602

**Published:** 2014-04-09

**Authors:** Wei Sun, Yuan Feng, Xu-Dong Yao, Yun-Fei Xu, Bo Peng, Min Liu, Jun-Hua Zheng

**Affiliations:** ^1^Department of Urological Surgery, Shanghai Tenth People's Hospital, Tongji University, Shanghai 200072, China; ^2^Department of Nephrology, Affiliated Drum Tower Hospital, Nanjing University, Nanjing, Jiangsu 210093, China

## Abstract

*Background.* Elevated urinary angiotensinogen (UA) was identified as novel prognostic biomarker capable of predicting chronic kidney disease, and in the present study, we will investigate the diagnostic value of UA in the patients of nephrolithiasis. *Methods.* Urine angiotensinogen levels and **α**1-microglobulin were measured by enzyme-linked immunosorbent assay (ELISA) in 60 patients presenting with nephrolithiasis and 50 sex- and age-matched healthy volunteers. Estimated glomerular filtration (eGFR) was calculated and, by simple regression analysis, the correlation of UA/**α**1-microglobulin levels and the decline of eGFR were analyzed as well. *Results.* Median UA levels was significantly increased in the nephrolithiasis patients compared with normal control (1250.78 ± 439.27 versus 219.34 ± 45.27 pg/mL; *P* < 0.01). The mean serum creatinine levels in patients with higher UA levels (>1250 pg/mL) was significantly higher than those with lower UA levels (<1250 pg/mL) [92.23 ± 18.13 **μ**mol/L versus 70.07 ± 11.17 **μ**mol/L; *P* < 0.05]. According to the single variate analysis, UA levels were significantly and positively correlated with urinary **α**1-microglobulin (*r* = 0.733; *P* = 1.33 × 10^−15^), while they were significantly and negatively correlated with eGFR (*r* = −0.343; *P* = 1.03 × 10^−4^). *Conclusion.* Urinary UA is a novel biomarker for patients with nephrolithiasis, which indicates renal tubular injury. Further study on the molecular pathogenic mechanism of UA and larger scale of clinical trial is required.

## 1. Introduction

Nephrolithiasis is a condition involving the development of stones in the kidney; it is a common disease with a prevalence of 5–8% worldwide [1]. The exact cause and etiology of nephrolithiasis remain unclear. The risk factors for developing nephrolithiasis include genetics, age, sex, geography, seasonal factors, diet, and occupations [2]. No specific predictive biomarker for the disease comes on the scene and many patients are diagnosed late after marked symptoms such as renal colic and hematuria appear. A reliable biomarker for nephrolithiasis which could predict earlier diagnosis, treatment, or better monitoring is greatly demanded.

Urine is supersaturated with oxalate ions and calcium which promotes crystals formation, and then crystals grow into larger ones; aggregation and retention of crystals are important factors of nephrolithiasis formation. During the procedure, the role of renin-angiotensin system (RAS) in kidney tissue was identified recently [3]. Moonen et al. reported that the levels of blood angiotensin-converting enzyme (ACE) were significantly increased in idiopathic hypercalciuric renal stone formers compared with healthy volunteers [4].

As already known, renin is made in the juxtaglomerular apparatus and released into the interstitial space, from where it may reach the circulation via diffusion across the peritubular capillaries. Proximal tubular fluid, however, also contains renin, suggesting that circulating renin is filtered in the kidney [5]. Circulating, liver-derived angiotensinogen (Ang) diffuses into the interstitium, reaching interstitial fluid levels that are comparable to those in blood [6]. Circulating ACE plays little, if any, role, and, thus, renal Ang II generation will depend entirely on locally expressed, membrane-bound ACE in the kidney [7]. Indeed, in human kidney, ACE is abundant in the brush border of the proximal tubule and, remarkably, usually absent in endothelial cells of any vessel type [8]. Endothelial neoexpression of ACE comes into play in different diseases, for example, diabetes mellitus and chronic arterial hypertension [9].

Recent interest focuses on the occurrence of both renin and angiotensinogen in urine, as markers of renal renin-angiotensin system (RAS) activity, potentially reflecting the disease state [10]. Elevated urinary angiotensinogen (UA) was identified as novel prognostic biomarker capable of predicting adverse outcomes in worsening of acute kidney injury and even need of hemodialysis therapy in intensive care unit [11]. However, there were no studies that examined in detail the urinary angiotensinogen in the patients of nephrolithiasis. In the present study, we compared urinary angiotensinogen levels between the patients with nephrolithiasis and healthy controls.

## 2. Materials and Methods

### 2.1. Patients

We conducted a case control study between January 2012 and March 2013 at Tongji University tenth peoples' hospital. Patients who were diagnosed as having nephrolithiasis during the period were enrolled in this study. Briefly, they were diagnosed by ultrasonography and radiography. No case was found by X-ray to have radiolucent stones or by clinical evaluation to have cystine or uric acid stones. If stone specimens were removed by surgery or obtained after medical treatment or shock-wave lithotripsy, composition of the stones was confirmed by infrared spectroscopy (Spectrum RX I Fourier Transform-Infrared System, Perkin-Elmer, USA) [12].

Normal controls were randomly selected from subjects receiving general health examinations at the same hospital during the same period. The controls had no past history of nephrolithiasis and no clinical findings of stones, which were confirmed by plain abdominal X-ray and abdominal ultrasound. Both cases and controls were excluded if they had a history of chronic urinary tract infection, renal failure, chronic diarrhea, gout, renal tubular acidosis, autoimmune diseases, primary and secondary hyperparathyroidism, and cancer. All study subjects were living in Shanghai. The study protocol was approved by the Institutional Review Board of Tongji University School of Medicine. Each subject provided signed informed written consent.

### 2.2. Samples Collection and Assays

Since the literature reported that urinary angiotensinogen significantly correlated with decline of estimated glomerular filtration (eGFR), thus we measured fasting serum levels of creatinine (Cr) in each patient (Wako Pure Chemical Industries, Osaka, Japan); and the eGFR was calculated by the following equations [13]:

(1) eGFR(mL/min⁡/1.73 m2)=194×Cr−1.094×age−0.287in  males,

(2) eGFR(mL/min⁡/1.73 m2)=194×Cr−1.094×age−0.287 ×0.739  in  females.

Urinary excretions of *α*1-microglobulin and angiotensinogen were measured with enzyme-linked immunosorbent assay (ELISA) kits, LZ Test Eiken *α*1-M (Eiken Chemical Co., Tokyo, Japan) and Human Total Angiotensinogen Assay Kit (Immuno-Biological Laboratories Co., Ltd., Fujioka, Gunma, Japan).

### 2.3. Statistical Analysis

Data obtained by measurements were given as mean ± standard deviation. Urinary levels of angiotensinogen, *α*1-microglobulin, and other parameters were compared by the Mann Whitney* U* test. The concentrations of urinary angiotensinogen were compared by Student's paired* t*-test. SPSS software (version 17.0, SPSS Inc., Chicago, IL, USA) was used for statistical analyses, and *P* < 0.05 was considered statistically significant.

## 3. Results

**Figure 1:**
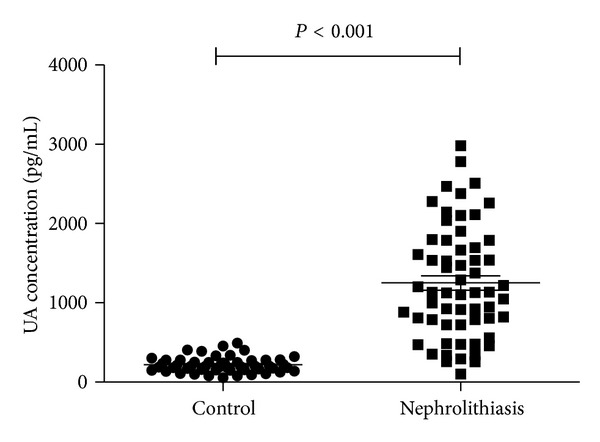
Urinary angiotensinogen (UA) concentration of nephrolithiasis patients and normal control. The UA concentration of nephrolithiasis patients was 1250.78 ± 439.27 pg/mL and was of significant statistical significance compared with the control group (219.34 ± 45.27 pg/mL; *P* < 0.001).

This study included 60 nephrolithiasis patients and 50 normal healthy people in the control group. The mean (±SD) of age of patients in the study and control groups was 45.5 ± 14.3 and 43.6 ± 12.7 years, respectively. The ratio of male/female was 37/23 in the study group and 35/15 in the control group. The demographic characteristics of both groups are summarized in Table 1. There were no significant differences in these parameters between patients and control. Calcium oxalate and calcium phosphate were the most prevalent in the nephrolithiasis patients (83%). Urine characteristics of both groups, including urine volume, pH, and uric acid, were listed in Table 1, as well. The mean urinary angiotensinogen levels were shown in the last line in Table 1. The UA concentration in nephrolithiasis patients was 1250.78 ± 439.27 pg/mL, which was significantly higher than in normal control group (219.34 ± 45.27 pg/mL; *P* < 0.01); the median 24 h urinary angiotensinogen excretion was significantly higher in nephrolithiasis patients versus normal control (8.7 ± 1.12 versus 4.1 ± 0.46 *μ*g/24 h; *P* < 0.01); urinary angiotensinogen-to-creatinine ratio was also significantly higher in nephrolithiasis patients versus normal control (18.9 ± 5.22 versus 4.3 ± 1.60 *μ*g/g; *P* < 0.01), as demonstrated in Table 1 and Figure 1.

**Figure 2:**
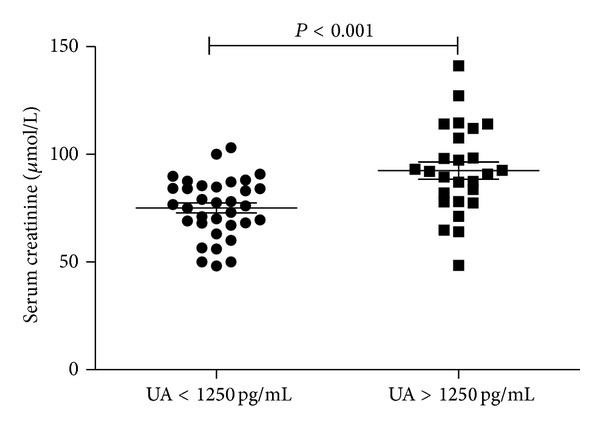
Serum creatinine levels in nephrolithiasis patients. The serum creatinine concentration of nephrolithiasis patients with UA < 1250 pg/mL was 70.07 ± 11.17 *μ*mol/L and was of significant statistical significance compared with nephrolithiasis patients with UA > 1250 pg/mL (92.23 ± 18.13 *μ*mol/L; *P* < 0.05).

To compare the potential effect of UA on the renal function, we divided the patients into two groups according to their UA levels: UA < 1000 pg/mL and UA > 1000 pg/mL, and we compared their mean serum creatinine and found that the serum creatinine levels with UA < 1250 pg/mL were 70.07 ± 11.17 *μ*mol/L; however, serum creatinine levels were significantly higher (92.23 ± 18.13 *μ*mol/L) in the nephrolithiasis patients with UA > 1250 pg/mL (*P* < 0.05), which was demonstrated in Figure 2.

**Figure 3:**
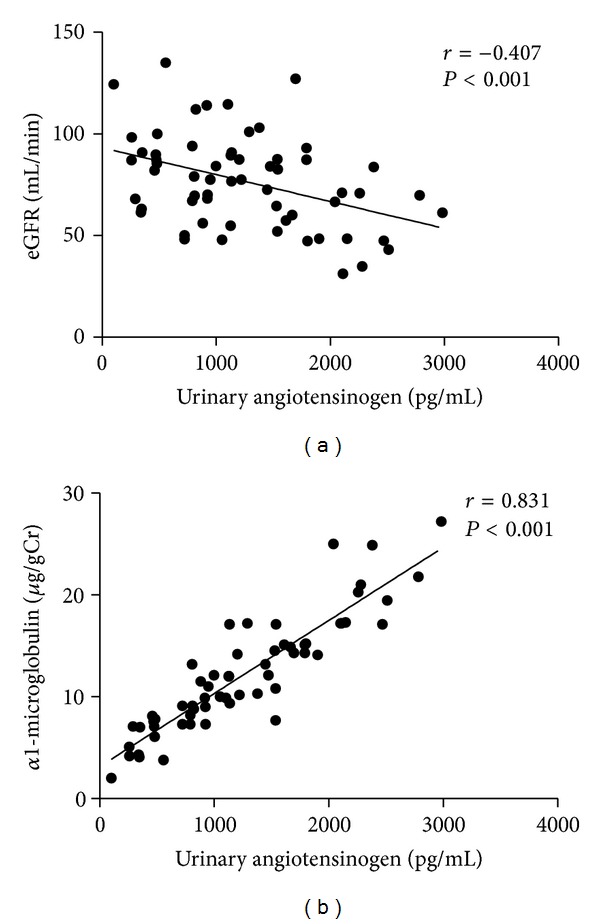
Simple correlation of urinary angiotensinogen concentration with eGFR and urinary *α*1-microglobulin in patients with nephrolithiasis (*n* = 60). (a) Estimated glomerular filtration ratio, eGFR (*r* = −0.407, *P* < 0.001); (b) urinary *α*1-microglobulin (*r* = 0.831; *P* < 0.001). Note: Spearman correlation coefficients were used.

By simple regression analysis of the parameters, urinary angiotensinogen levels were significantly and positively correlated with urinary *α*1-microglobulin (*r* = 0.733; *P* = 1.33 × 10^−15^), while they were significantly and negatively correlated with eGFR (*r* = −0.343; *P* = 1.03 × 10^−4^) (Table 2 and Figure 3).

## 4. Discussion

Our study investigated the urinary angiotensinogen levels in nephrolithiasis patients and initially found that the UA concentrations in the patients with nephrolithiasis were significantly higher than those in normal subjects. In spite of normal renal function parameters in nephrolithiasis patients, the mean serum creatinine levels were significantly higher in patients versus normal control. In addition, we performed a simple regression analysis and demonstrated that UA levels significantly and positively correlated with urinary *α*1-microglobulin and UA levels significantly and negatively correlated with eGFR, which suggests potential renal tubular injury in nephrolithiasis patients.

In the past studies, it has been suggested that urinary excretion of angiotensinogen reflects intrarenal angiotensin II levels [14, 15]. Angiotensin II is a central mediator of renal injury because of its ability to produce glomerular capillary hypertension that results in damage to glomerular epithelial, endothelial, and mesangial cells [16]. Furthermore, angiotensin II and aldosterone have several nonhemodynamic effects that are also important in the pathogenesis of chronic kidney disease, including activation of pathways associated with inflammation, fibrosis, extracellular matrix accumulation, reactive oxygen species, and endothelial dysfunction [17]. As already known, the etiology of renal calculi is closely related to acute/chronic renal tubular injuries and subsequent crystal deposition and Randall's plaque formation. However, whether UA is elevated or decreased in kidney stone patients, no relative studies emerged until our present investigation.

In Alge et al.'s study, urinary angiotensinogen could be useful as a prognostic acute kidney injury biomarker in the setting of the intensive care unit; in a recent study that used a definition of prerenal azotemia, which was very similar to Alge et al.'s, cystatin C, neutrophil gelatinase-associated lipocalin (NGAL), IL-18, and kidney injury molecule 1 (KIM)-1 were elevated in ICU patients with prerenal AKI compared to those without AKI but were lower than values for patients whose AKI did not resolve within 48 hrs [18]. An important limitation of our study is that it was a relatively small retrospective biomarker qualification study with 60 nephrolithiasis patients. Larger studies will be needed to confirm the prognostic ability of angiotensinogen in this population.

In humans, plasma angiotensinogen reaches urine via glomerular filtration, like albumin, and the normal urinary angiotensinogen levels in humans are ~0.2 pmol/mL versus ~1,200 pmol/mL in plasma. Thus, the urinary angiotensinogen levels range from 0.01% to 0.1% of the plasma levels in humans. In contrast, urinary angiotensinogen levels in rodents are much higher and range from 0.1 to 400 pmol/mL, implying that the urinary angiotensinogen levels in rodents are sometimes higher than their plasma levels [19]. Less than 100-fold higher urinary angiotensinogen levels in rodents suggested the concept that urinary angiotensinogen is exclusively plasma-derived in humans, whereas it reflects angiotensinogen release from renal tissues, possibly proximal tubules, in rodents [20].

Urinary *α*1-microglobulin is filtered freely through glomerular capillaries and reabsorbed by the proximal tubules [21]. Thus, urinary *α*1-microglobulin is a marker for proximal tubule dysfunction, and increased levels of urinary *α*1-microglobulin have been reported in patients with type 1/2 diabetes [22]. The assessment of proximal tubule dysfunction by urinary *α*1-microglobulin allows the early diagnosis of diabetic nephropathy prior to the appearance of microalbuminuria and also predicts the progression of diabetic nephropathy. There is little literature about *α*1-microglobulin in nephrolithiasis patients, however [23]. Plasma angiotensinogen is filtered through glomerular capillaries, and urinary angiotensinogen is mainly derived from plasma. Subsequently, urinary angiotensinogen is largely removed via endocytotic uptake in tubules in a megalin-dependent manner. Endocytotic angiotensinogen is subsequently degraded and the contents of angiotensinogen in the proximal convoluted tubules highly correlated with plasma levels of angiotensinogen [24]. Thus, previous studies also support the idea that urinary excretion of angiotensinogen reflects not only abnormalities of glomerular filtration barrier but also proximal tubular functions. It has been reported that urinary angiotensinogen levels increase before glomerular injuries in the patients as well as in rodents [25]. Another limitation of our study is that no detailed data about hemodynamic index such as fractional excretion of sodium (FENa), perfusion flow rate (PFR), and even podocyte function parameters should be taken into account in future studies. As the literature reported, angiotensinogen was expressed in proximal tubular cells, and UA excretion was correlated with renal Ang II but not plasma Ang II; it was suggested that UA reflects renal Ang II production. Ang II will stimulate renal angiotensinogen synthesis, resulting in both elevated renal angiotensinogen levels and increased urinary angiotensinogen excretion, thus potentially creating a positive feed-forward loop. According to this concept, the rise in renal Ang II content following Ang II infusion involves de novo Ang II formation in the kidney from locally generated angiotensinogen [26]. The precise mechanism for the increment of renal de novo angiotensinogen generation in nephrolithiasis patients should be investigated in further studies.

In conclusion, we conclude in our study measurement of UA was a novel and useful marker for patients with nephrolithiasis. The investigation of UA suggests urologists to concern about previous neglected tubular injury in clinical kidney stone patients and greatly enhances our understanding of tubular injury in etiology of nephrolithiasis. Further validation of serum creatinine and urinary *α*1-microglobulin shows us the disease progression in nephrolithiasis cohort, which might require multicenter randomized study.

## Figures and Tables

**Table 1:** General characteristics of nephrolithiasis patients and healthy controls.

Factor	Nephrolithiasis (*n* = 60)	Healthy controls (*n* = 50)
Age (years)	45.5 ± 14.3	43.6 ± 12.7
Gender (male/female)	37/23	35/15
Body weight (kg)	62.68 ± 11.86	65.64 ± 9.52
Height (m)	1.68 ± 0.10	1.70 ± 0.08
Body mass index (kg/m^2^)	22.58 ± 5.97	23.05 ± 4.29
Urine volume (mL/24 h)	1487 ± 531.8	1158 ± 498.7
Specific gravity	1021 ± 7.8	1023 ± 8.4
Urine creatinine (g/24 h)	1.7 ± 0.8	1.6 ± 0.9
Urine pH	6.21 ± 0.56	6.09 ± 0.72
Urine uric acid (mg/24 h)	667 ± 153	679 ± 144
Urine angiotensinogen (pg/mL)	1250.78 ± 439.27*	219.34 ± 45.27
24 h UA excretion (*μ*g/24 h)	8.7 ± 1.12*	4.1 ± 0.46
UA/urine creatinine ratio (*μ*g/g)	18.9 ± 5.22*	4.3 ± 1.60

*P < 0.01 in patients compared with healthy controls.

**Table 2:** Simple correlation of urinary angiotensinogen with various clinical parameters in patients with nephrolithiasis (*n* = 60).

	Angiotensinogen (pg/mL)
Age (years)	*r* = −0.041; *P* = 0.754
BMI (kg/m^2^)	*r* = −0.080; *P* = 0.517
SBP (mmHg)	*r* = −0.031; *P* = 0.815
DBP (mmHg)	*r* = −0.182; *P* = 0.127
Cr (µmol/L)	*r* = 0.261; *P* = 0.021*
UN (µmol/L)	*r* = 0.397; *P* = 0.002**
Urine uric acid (mg/24 h)	*r* = 0.077; *P* = 0.538
*α*1-microglobulin (µg/gCr)	*r* = 0.831; *P* < 0.001**
eGFR (mL/min)	*r* = −0.407; *P* < 0.001**

*P < 0.05; **P < 0.01. Spearman correlation coefficients are used.

BMI: body mass index; Cr: serum creatinine; DPB: diastolic blood pressure; eGFR: estimated glomerular filtration ratio; SBP: systolic blood pressure; UN: serum urea nitrogen.

## Abbreviations

UA: Urinary angiotensinogen
ACE: Angiotensin-converting enzyme
RAS: Renin-angiotensin system
Ang: Angiotensinogen
eGFR: Estimated glomerular filtration
Cr: Creatinine
NGAL: Neutrophil gelatinase-associated lipocalin
AKI: Acute kidney injury
CKD: Chronic kidney disease
KIM: Kidney injury molecule
ICU: Intensive care unit
ELISA: Enzyme-linked immunosorbent assay
FENa: Fractional excretion of sodium
PFR: Perfusion flow rate.
